# Sodium New Houttuyfonate Affects Transcriptome and Virulence Factors of *Pseudomonas aeruginosa* Controlled by Quorum Sensing

**DOI:** 10.3389/fphar.2020.572375

**Published:** 2020-10-02

**Authors:** Yeye Zhao, Longfei Mei, Yuanqing Si, Jiadi Wu, Jing Shao, Tianming Wang, Guiming Yan, Changzhong Wang, Daqiang Wu

**Affiliations:** ^1^Department of Pathogenic Biology and Immunology, College of Integrated Chinese and Western Medicine, Anhui University of Chinese Medicine, Hefei, China; ^2^Key Laboratory of Chinese Herbal Compound Formula in Anhui Province, Anhui University of Chinese Medicine, Hefei, China; ^3^Division of Molecular and Cell Biophysics, Hefei National Science Center for Physical Sciences, University of Science and Technology of China, Hefei, China

**Keywords:** *Pseudomonas aeruginosa*, virulence factors, biofilm, quorum sensing, sodium new houttuyfonate, transcriptome

## Abstract

As a major opportunistic pathogen, *Pseudomonas aeruginosa* can produce various virulence factors and form biofilms. These processes are controlled by the quorum sensing (QS) system. Sodium new houttuyfonate (SNH) is an adduct of houttuyfonate, the main component of the common Chinese medicine plant Houttuynia cordata, which has antibacterial and anti-inflammatory effects. We evaluated the effect of SNH on *P. aeruginosa* biofilms, virulence factors, and transcription. Transcriptome analysis showed that the key *rhlI* and *pqsA* genes of the *P. aeruginosa* QS system were down-regulated after SNH treatment. SNH reduces proteases and pyocyanin production and inhibits biofilm formation by regulating the *P. aeruginosa* QS system. SNH also changes the expression of genes related to virulence factors and biofilms (*lasA, lasB, lecA, phzM, pqsA*, and *pilG*). These results suggested that the mechanism of SNH against *P. aeruginosa* by affecting the expression of biofilm and virulence factors controlled by quorum sensing.

## Introduction

*Pseudomonas aeruginosa* is a common clinical opportunistic pathogen (Denning et al., 2003; Xiaoming et al., 2012; Gurunathan et al., 2014). Patients with severe burns (Ramsey and Wozniak, 2005; Bentzmann and Patrick, 2011) or metabolic diseases are sensitive to infection when the body resistance decreases. *P. aeruginosa* infections has a high morbidity and mortality in the clinic because of its secretion of a large number of virulence factors and increasing antibiotic resistance (Breidenstein et al., 2011). Therefore, there is an urgent need to develop new drugs against *P. aeruginosa* infection.

Bacterial quorum sensing (QS) is a cellular communication mechanism that depends on the concentration of signal molecules (Ma et al., 2012; Asfahl and Schuster, 2017). There are main three QS regulation systems in *P. aeruginosa* (Kim et al., 2015), the Las, Rhl, and PQS systems (Schuster et al., 2013; Kalia et al., 2015; Rajkumari et al., 2018), whose transcription regulators are LasR, RhlR, and PqsR respectively. The *P. aeruginosa* QS system is involved in regulating the expression of virulence factors and biofilms (Van Delden and Iglewski, 1998; Williams and Camara, 2009). At an appropriate concentration, these receptor proteins bind to their respective signal molecules and induce transcription encoding different virulence products such as elastase, rhamnolipid, and pyocyanin. The LasI/Rhl system contains the synthase LasI/RhlI and the transcription activator LasR/RhlR. The Las system can synthesize and recognize N-3-oxo-C12-HSL through LasI synthetase and transcription regulator *LasR*. The second AHL system is the Rhl system, which can synthesize C4-HSL. The combination of RhlR and C4-HSL activates the expression of antibiotics, rhamnolipids, alkaline proteases, elastase, lectin LecA, cyanide, and genes produced by mass movement. Therefore, it is important to understand the relationship between antibacterial drugs and the *P. aeruginosa* QS system.

**Figure 1:**
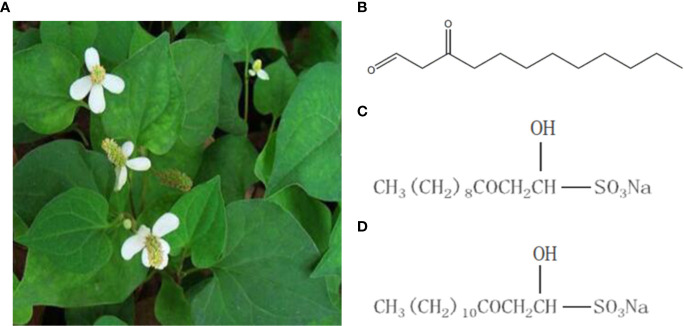
*Houttuynia cordata* Thunb and related compounds. **(A)** *Houttuynia cordata* Thunb (www.cqcqfn.com). **(B)** Houttuynin. **(C)** Sodium houttuyfonate and **(D)** Sodium new houttuyfonate.

*Houttuynia cordata* is a traditional Chinese medicine, which is derived from Saururaceae. *H. cordata* has the functions of antibacterial, antiviral, improving immunity, diuresis and so on. And it is one of the varieties officially identified medicine and food by the Ministry of Health **(****Figure 1****)**. Sodium new houttuyfonate (SNH) is a sodium bisulfite adduct of the active ingredient of the Chinese herbal medicine *H. cordata* (Shao et al., 2012) and dodecanoylacetaldehyde. SNH has spectrum wide antibacterial activity, few adverse reactions, and improves immunity (Juanjuan, 2009; Yumei et al., 2012; Yang et al., 2016). Previously, we confirmed that sodium houttuyfonate (SH) has a certain inhibitory effect on *P. aeruginosa* (Shao et al., 2013; Wu et al., 2014; Wu et al., 2015). SNH and SH structures are highly similar, but SNH is more chemically stable than does SH (Yumei et al., 2012). Therefore, it is important to understand the inhibitory effects and mechanisms of SNH on *P. aeruginosa*. We speculate that SNH may also inhibit the activity of *P. aeruginosa* by regulating the QS system.

## Materials and Methods

### Chemicals, Bacteria, and Culture Medium

SNH was purchased from Shanghai Yuanye Biological Technology Co., Ltd. (Shanghai, China), with the content ≥ 98%. Azithromycin (AZM) was acquired from the Northeast Pharmaceutical Group Shenyang No. 1 Pharmaceutical Co., Ltd. (Shenyang, China). *P. aeruginosa* strain ATCC 27853 was purchased from the China National Institute for the Control of Pharmaceutical and Biological Products (Beijing, China). PDP medium was acquired from Qingdao Hi-Tech Park Haibo Biotechnology Co., Ltd. (Qingdao, China). A single ATCC 27853 colony was selected from Luria-Bertani (LB) solid medium (Hangzhou, China) and cultured in 5 ml LB liquid media (Hangzhou, China) at 37°C, with shaking at 220 r/min for 24 h, then by centrifugation at 10,800 g for 1 min. The supernatant was discarded and the pellet was resuspended in sterile saline solution for optic density detection at 600 nm(OD600) in a UV spectrophotometer. The absorbance of cell suspensions was adjusted to 0.08–0.1, and diluted to 10^7 times for later use for further experiments.

### Measurement of Minimum Inhibitory Concentration and Minimum Bactericidal Concentration

The minimum inhibitory concentration (MIC) (Wiegand et al., 2008) and minimum bactericidal concentration (MBC) of SNH were determined using the microdilution method. Different concentrations of SNH solution (100 μl; 2,048, 1,024, 512, 256, 128, 64, 32, 16, 8, and 4 μg/ml) were added with an equal volume of diluted bacterial solution to a 96-well plate. AZM treatment was used as a positive control and culture medium and bacterial solution were used as a blank control group. Plates were incubated at 37°C for 24 h, and the minimum drug concentration with aseptic growth was taken as the MIC. An aliquot (100 μl) was transferred from the sterile growth well to the nutrient agar plate, and cultured at 37°C. After 24 h, MBC was determined as less than 5 colonies on the plate. The experiment was repeated three times.

### Growth Curve

SNH solutions (100 μl) were prepared to final concentrations of 2,048, 1,024, and 512 μg/ml. Equal volumes of SNH bad bacterial solutions were added to 96-well plates, and the control groups established. Plates were incubated at 37°C, the OD_600_ (Nalca et al., 2006) measured at different time points, and the growth curves were drawn.

### Drug Treatment and Sample Collection

SNH (2 ml) and an equal volume of bacterial solution was added to the test tube to obtain a concentration of 1 x MIC SNH and the bacteria was cultured at 37°C for 24 h. The blank control group was the SNH treatment control group and no drugs were added. Centrifuge to remove supernatant, and rinsed three times with sterile water. The collected bacterial sample was placed in a centrifuge tube, sealed with sealing film, and placed directly onto dry ice to perform RNA-seq (Poluri et al., 2019). For each group, four samples were prepared under the same experimental conditions.

### Transcriptome Sequencing

SNH-induced sequencing of *P. aeruginosa* transcription was performed using the BGISEQ-500 platform (Huada Gene Technology). First, total RNA was processed and purified, and reverse transcription performed to synthesize cDNA which was then used to produce double-stranded DNA. The end of the synthesized double-stranded DNA was flattened and phosphorescence added to the 5′ end. The 3′ end then forms a sticky end protruding from an “a”, and a bubbly joint with a protruding “t” at the 3′ end is connected. PCR amplification is then performed using specific primers, PCR products thermally denatured into single strands, and the single-stranded DNA is circularized to obtain a single-stranded circular DNA library.

Data are obtained by transcription sequencing was counted using the SOAPnuke (Yuxin et al., 2018) filtering software and filtered using trimmomatic (Bolger et al., 2014). First, reads containing the linker, with unknown base N content greater than 5%, and those of low-quality were removed. NC_002516.2 was used as reference genome. Bowtie2 software was used to align clean reads to the reference gene sequence, and RSEM (Dewey and Bo, 2011; Langmead and Salzberg, 2012) was used to calculate expression levels.

### Differential Gene Expression Analysis

Differentially expressed genes (DEGs) were detected based in the method described by Wang et al. (2010). For comparison, the *P*-values calibrated for multiple tests were performed according to the Benjamini and Hochberg method. False discovery rate (FDR) threshold ≤ 0.05 and | log_2_fold change (FC)| >1 were defined as significant DEGs (Sun et al., 2015).

### Gene Ontology and Kyoto Encyclopedia of Genes and Genomes Enrichment Analysis

DEGs were subjected to Gene Ontology (GO) enrichment analysis and Kyoto Encyclopedia of Genes and Genomes (KEGG) pathway analysis (Sun et al., 2017). In general, DEGs are annotated into GO entries, and the number of genes under each entry is counted to analyze GO and KEGG pathway enrichment. GO entries and KEGG pathways are corrected for *P*-value by FDR, and the *Q*-value ≤ 0.05 is considered significantly enriched. The major biological functions that DEGs perform can be evaluated by analysis of significant GO and KEGG pathway enrichment.

### Determination of Total Protease, LasA Staphylococcal Protease, LasB Elastase, LecA Lectin, Pyocyanin, and Floating Swimming Ability

Bacterial supernatants (500 μl) extracted following treatment with different SNH concentrations were incubated with 1 ml of skim milk (1.25%) at 37°C for 20 min, and their OD_600_ measured (El-Mowafy et al., 2014). The overnight *Staphylococcus aureus* culture was boiled for 10 min, centrifuged for 10 min at 10,000 g, and the precipitate was resuspended in 10 mM with Na_2_HPO_4_ (pH4.5) to an OD_600_ of 0.8. The supernatants with different SNH concentrations and *S. aureus* suspensions were mixed at a ratio of 1:9, and OD_600_ was measured at different time points to calculate the activity of LasA Staphylococcus protease (Kessler et al., 1993). Bacterial supernatants with different SNH concentrations were used to measure the amounts of secreted elastase LasB and lectin LecA using ELISA kits (Shanghai, China). Different concentrations of pyocyanin assay medium, PAO1, cultured at 37°C for 24 h at 220 r/min were removed from the shaker and centrifuged for 1 min at 10,800 g to remove the supernatant, diluted with sterile water to 0.5 M colorimetric tube, and diluted to 10^4^ times for later use, inoculated on the slant of the medium, and cultured at 37°C for 24 h. Chloroform (3 ml) was added to the test tube, mixed thoroughly and allowed to stand for 15 min. When the chloroform layer turned green they were transferred to another test tube. Then 1 ml of 1 mol/L HCL was added, mixed, and left to stand for 5 min. The OD_520_ was measured when the solution turned pink. The pyocyanin content was determined by: OD_520_ * 17.072 (Kong et al., 2005). In a word, the supernatant samples were tested for virulence factors 24 h after coincubation with bacteria and drugs, the expression was standardized by growth OD_600_. Floatation exercise plates with different drug concentrations (10 g/L tryptone, 10 g/L NaCl, 0.3% agar) were prepared. The strain cultured for 24 h at 37°C and 220 r/min was taken from the shaker and the supernatant was centrifuged at 12,000 r/min, diluted to 0.5 M, and serially diluted 100 times. Using a pipette gun, 2 μl of bacterial liquid was inoculated onto the surface of the floating motion medium, and was placed at 37°C for 24 h to measure colony diameter (Rashid and Kornberg, 2000).

### Biofilm Formation

SNH solution (2 ml) with final concentrations of 2,048, 1,024, and 512 μg/ml and an equal volume of diluted bacterial solution were added to a 6-well plate and placed a sterile cover glass. The positive control AZM and blank control group were set up simultaneously. Plates were incubated at 37°C for 24 h, then the planktonic bacteria were washed 3 times on the slide with PBS. The samples were then placed in pre-cooled 2.5% glutaraldehyde solution at least 2 hours in the dark at 4°C, and placed in 30%, 50%, 70%, 90% and 100% ethanol for gradient dehydration for 10 minutes. After drying at room temperature, the biofilm samples were sprayed gold after vacuum drying, and observed and imaged (×5000) with Scanning Electron Microscope (GeminiSEM 500, Germany). Crystal violet (0.4%) was used for staining, 30% acetic acid was used for dissolution, and absorbance was measured at 540 nm as previously described (Corral-Lugo et al., 2016).

### RT-PCR and qRT-PCR

Expression of *rhlI, pqsA, lasA, lasB, lecA, phzM*, and *pilG* genes in the QS system was evaluated using PCR. Preparations of different concentrations of bacteria and drugs were used for coculture. In the control group, Mueller-Hinton Broth (MH (B)) medium was added instead of SNH. After culturing at 37°C for 24 h, the supernatant was removed and total RNA extracted from the bacteria using TIANGEN′s RNAprep Pure Cell/Bacteria Kit and following the manufacturer′s instructions. cDNA was reverse transcribed using TIANGEN′s FastQuant RT Kit. RT-PCR was performed using 2 x EasyTaq PCR SuperMix (TransGen Biotech, Beijing, China) and a GENETEST Series Gene Amplifier (Hangzhou, China). The reaction involved 35 amplification cycles of: 94°C 5 min, 94°C 30 s, 56°C 30 s, 72°C 30 s, and 72°C 10 min. The synthesized DNA was electrophoresed on a 1.5% agarose gel, and then imaged on a gel imager. SYBR dye was used for qRT-PCR experiments and the 25 μl reaction system included 12.5 μl SYBR dye, 1 μl upstream primer and 1 μl downstream primer (10 μM), 0.5 μl cDNA, and 11 μl DEPC water. The reaction was performed on an Applied Biosystems 7,500 real-time PCR system (USA) and the reaction steps were: 95°C 15 s, 56°C 30 s, 72°C 30 s, for 40 amplification cycles. Reactions were gradient cooled at 95°C–60°C and the dissolution curve detected. *rpoD* was used as an internal reference, and the gene expression determined by 2^-Δ Δ CT^. Primer sequences are shown in **Table 1**.

**Table 1:** Sequences of primers used in this study.

Primer name	DNA chain	Sequence (5′-3′)
*lasR* (Wu et al.,2014)	Forward Reverse	CATCGTCGCAACTACCC GCGCACCACTGCAACACT
*lasI* (Wu et al.,2014)	Forward Reverse	TTGCTCGCCGCACATC GGCACGGATCATCATCTT
*rhlI* (Wu et al.,2014)	Forward Reverse	ATCCGCAAACCCGCTAC GCAGGCTGGACCAGAATAT
*lasA* (Wu et al.,2014)	Forward Reverse	CTACAGCATCAACCCGAAAG TAGCGCCGCGACAACT
*lasB* (Wu et al.,2014) *lecA* (Hickey et al.,2018) *phzM* (Wu et al.,2014)	Forward Reverse Forward Reverse Forward Reverse	GTTCTATCCGCTGGTGTCG CGCTGCCCTTCTTGATG TGCGCTGGTCATGAAGAT TG GAACGAGCCGGAGTTATTGC GACATGGTGCTGTTCTACGG TGGAATGCCAGGTTGCTC
*pilG* (Wu et al.,2014) *pqsA* (Wu et al.,2014) *rpoD* (Wu et al.,2014)	Forward Reverse Forward Reverse Forward Reverse	ACGGTTTGAAAGTGATGGTG AAATGATGTTCGGATGGGT TGGTGGTGCGTGAAGCC GGAACCCGAGGTGTATTGC AGGCCGTGAGCAGGGAT GGTGGTGCGACCGATGT

### *In Vivo *Evaluation of Efficacy of SNH Against* P. aeruginosa*

*Galleria mellonella* larvae were obtained from Tianjin Huiyude Biological Technology Co., Ltd., with size of 2-3 cm and weight of 0.2-0.3 g. The larvae were stored at 4°C for later use. SNH and AZM were dissolved in PBS without any cosolvent to avoid the possible toxic effects of cosolvent against larvae. The treatment groups of larvae were set as follows: PBS group (without any drugs and infection, infection (*P. aeruginosa* 10^4 CFU per one larvae, the rest of infection groups were treated with same concentration of *P. aeruginosa*) + PBS group, infection + SNH 2048 μg/ml group, infection + SNH 1024 µg/ml group, infection + SNH 512 µg/ml group, infection + AZM 64 µg/ml group. The vivo assays were carried as previously described (Wu et al., 2020).

### Statistical Analysis

SPSS 23.0 statistical software was used for analysis, and the results are reported as mean ± standard deviation. All procedures were performed in triplicate.

## Results

### Effect of SNH on *P. aeruginosa* Growth

We evaluated the antibacterial potential of SNH against *P. aeruginosa* and determined its MIC and MBC. At a concentration of 2048 μg/ml, aseptic growth is visible to the naked eye, indicating that the MIC of SNH to *P. aeruginosa* is 2048 μg/ml. These results show that SNH has certain antibacterial potential against *P. aeruginosa*. MBC was evaluated based on MIC results, and the bactericidal activity of SNH under MIC was observed. By counting observing plated ATCC 27853 bacteria, it was found that all long bacteria were long and that the number of colonies was > 5. The MBC results suggested that SNH had a bacteriostatic effect on ATCC 27853 and not a bactericidal effect. Therefore, the antibacterial effect of SNH is suitable for use against infections caused by *P. aeruginosa*.

**Figure 2:**
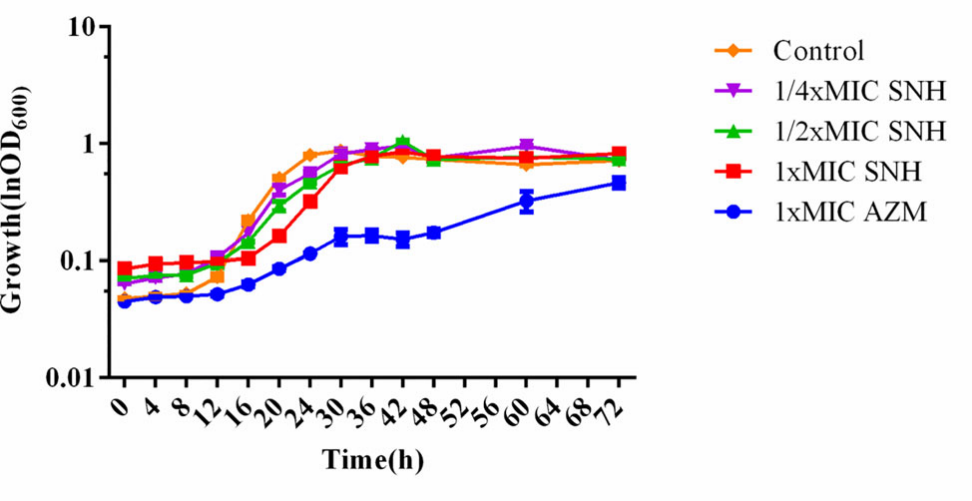
Growth curves of *P. aeruginosa* treated with different concentrations SNH.The drug concentration of treatments was as follows: Control (without any drugs), 512 μg/ml (1/4×MIC) SNH, 1,024 μg/ml (1/2×MIC) SNH, 2,048 μg/ml (1×MIC) sodium new houttuyfonate (SNH), and 64 μg/ml (1×MIC) azithromycin (AZM).

SNH had no effect on the growth of *P. aeruginosa* below the MIC (**Figure 2**). Compared with the untreated blank control, the OD value decreased in the presence of SNH before 24 h, but there was no statistical difference (Data not shown), and OD_600_ decreased slightly after 30 h, but compared with AZM group, the data showed significant difference, and AZM group decreased significantly from 36 h to 42 h indicating that SNH did not affect the growth of *P. aeruginosa*, and the change of biofilm formation was not caused by the change of growth.

### Transcriptome Sequencing and Clustering DEGs

**Figure 3:**
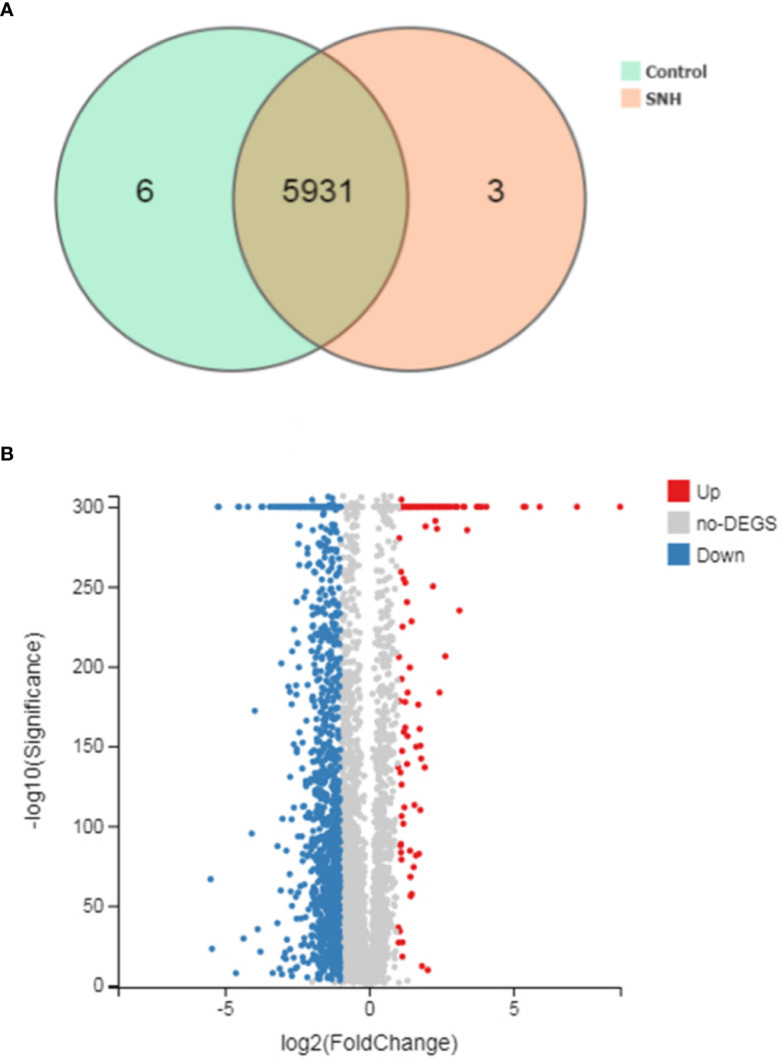
**(A)** Venn picture of differentially expressed genes (DEGs) and **(B)** Volcano graph between sodium new houttuyfonate (SNH) treated group and control group.

To further understand the antibacterial mechanism of SNH, we performed transcriptome sequencing. Totally 170.44 MB of transcriptome sequencing data was obtained after RNA-seq applying BGISEQ-500 platform. The original sequencing sequence data has been deposited into the NCBI SRA database (SRA accession: PRJNA542020) (http://identifiers.org/ncbi/insdc.sra:SRP197195.). In total, 5940 reference genes (https://www.ncbi.nlm.nih.gov/geo/query/acc.cgi?acc=GSE133428.) were assembled from sequencing data, with 5,937 (99.95%) known genes in the control group and 5,934 (99.9%) in the SNH treated group. Principal component analysis was based on FDR threshold ≤ 0.05 and | log_2_ fold change (FC) | > 1, and 1,674 significant DEGs were identified. Of these DEGs, 1,454 were down-regulated and the remaining 220 were up-regulated (**Figure 3**).

### Analysis of GO and KEGG Pathway of DEGs

**Figure 4:**
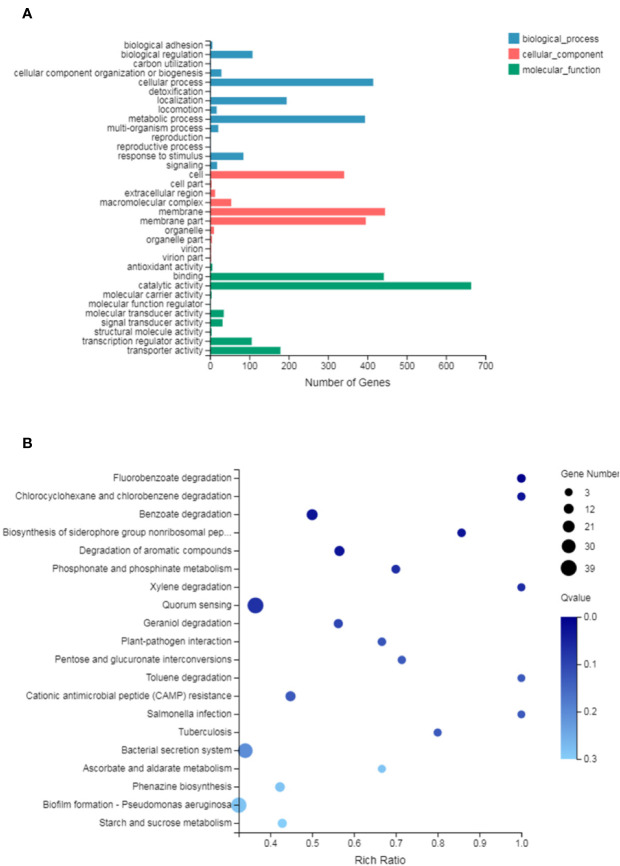
**(A)** Differentially expressed genes (DEGs) of biological processes, cellular component and molecular function Gene Ontology (GO) terms. **(B)** Statistical enrichment of DEGs in Kyoto Encyclopedia of Genes and Genomes (KEGG) pathways.

GO analysis provides reliable gene product descriptions from various databases and offers a set of dynamic, controlled, and structured terminologies to describe gene functions and products in organism. According to GO functions, all DEGs were classified into: biological process, cellular component, and molecular function. The most annotated functions were “catalytic activity” (GO: 0003824), “membrane” (GO: 0016020), “binding” (GO: 0005488), “cellular process” (GO: 0009987), “membrane part” (GO: 0044425), “metabolic process” (GO: 0008152), and “cell” (GO: 0005623). There were 664 genes in “catalytic activity”, 445 genes in “membrane”, 442 in “binding”, 396 in “membrane part”, 415 in “cell process”, and 294 in “metabolic process” (**Figure 4A**). DEGs were also enriched in KEGG pathways, which provide data on biological systems and their relationships at molecular, cellular, and organism levels. The KEGG pathways were annotated using the assembled *P. aeruginosa* transcriptome, and the results were mapped with GO terms. The first three functions that were most significantly enriched in KEGG pathway analysis were the QS system, biofilm formation, and bacterial secretion system Type I, Type II, Type III, and Type VI was changed under SNH treatment (**Supplementary Material Figure 2**). Among them, 39 genes were enriched in the QS system, 37 genes were enriched in biofilm formation, and 34 genes were enriched in the secretion system. In addition, degradation of fluorobenzoic acid, degradation of chlorocyclohexane and chlorobenzene, degradation of benzoic acid, and biosynthesis of non-ribosomal peptides containing iron carriers are the most significantly abundant signal pathways (**Figure 4B**). These results show that *P. aeruginosa* gene expression was up-regulated or down-regulated after SNH treatment compared with the control group. These results indicate that there are certain interactions between SNH and *P. aeruginosa*, and suggest that SNH may further inhibit the expression of virulence factors and the biofilm formation by influencing the QS system and bacterial metabolic pathways.

### Effect of SNH on *P. aeruginosa* DEGs

To better understand *P. aeruginosa* transcription changes under SNH treatment conditions, genes with expression differences were screened using GO and KEGG analyses. Expression genes involved in biofilm formation, the QS system, and phenazine biosynthesis were selected and examined further to reveal the effect of SNH on *P. aeruginosa*, and to classify their relative expression changes. The results show that among the 32 genes related to biofilm formation, QS system, and phenazine synthesis, 10 were up-regulated and 22 were down-regulated (**Table 2**), suggesting that SNH may have an inhibitory effect on the production of related virulence factors.

**Table 2:** Genes differentially expressed under sodium new houttuyfonate (SNH) and sodium houttuyfonate (SH) treatment.

Gene name	log2FoldChange		Category
	SNH	SH	
*phnA/trpE*	–2.32	–2.62	Biofilm
*phnB/trpG*	–1.43	–1.12	Biofilm
*pqsA*	–2.93	–3.78	Phenazine, Biofilm
*pqsB*	–3.34	–3.75	Phenazine, Biofilm
*pqsC*	–2.76	–3.15	Phenazine, Biofilm
*pqsD*	–2.91	–3.24	Phenazine, Biofilm
*pqsE*	–2.89	–3.05	Phenazine, Biofilm
*lasI*	/	–2.44	Biofilm
*rhlI*	–1.11	–1.5	Biofilm
*rhlA*	–1.87	–3.05	Biofilm
*rhlB*	–1.15	–1.61	Biofilm
*rhlC*	–1.15	–1.16	Biofilm
*lecA*	–2.8	–4.02	Biofilm
*hsbR*	–1.85	–1.88	Biofilm
*hsbA*	–1.4	–1.26	Biofilm
*roeA*	–1.58	–1.02	Biofilm
*mucR*	–1.03	–1.53	Biofilm
*pslB*	1.02	1.72	Biofilm
*pslL*	1.19	1.66	Biofilm
*pslJ*	1.08	1.37	Biofilm
*pslI*	1.13	1.44	Biofilm
*pslH*	1.26	1.62	Biofilm
*pslG*	1.25	1.64	Biofilm
*pslF*	1.2	1.62	Biofilm
*pslE*	1.16	1.65	Biofilm
*pslD*	1.16	1.51	Biofilm
*rfbN*	1.04	1.23	Biofilm
*algA*	1.0	1.19	Biofilm
*alg44*	–1.3	–1.46	Biofilm
*phzI*	–1.1	/	Quorum Sensing
*lasB*	–1.6	/	Quorum Sensing
*GadC*	–1.14	/	Quorum Sensing
*LsrG*	–1.1	/	Quorum Sensing
*TofI*	–1.1	/	Quorum Sensing

### Effect of SNH on *P. aeruginosa* Virulence Factor

**Figure 5:**
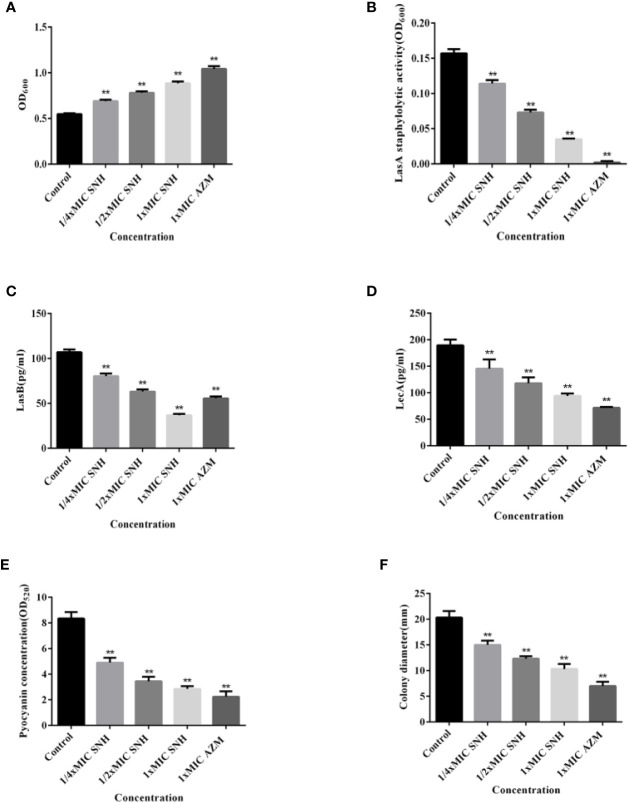
Effect of sodium new houttuyfonate (SNH) on virulence factor of *P. aeruginosa*. **(A)** Total protease. **(B)** LasA staphylococcal protease. **(C)** LasB elastase. **(D)** LecA lectin. **(E)** Pyocyanin. **(F)** Float swimming. The drug concentration of treatments was as follows: Control, 512 μg/ml (1/4×MIC) SNH, 1024 μg/ml (1/2×MIC) SNH, 2,048 μg/ml (1×MIC) SNH, and 64 μg/ml (1×MIC) azithromycin (AZM). Data represent the means ± SD (n=3, **p < 0.01 vs. control group).

The total protease, LasA staphylococcus protease, LasB elastase, LecA lectin, and pyocyanin levels and swimming ability were assessed (**Figure 5**). Total protease, LasA staphylococcal protease, LasB elastase, LecA lectin, and pyocyanin production, along with swimming ability, were all inhibited. This inhibition was dose-dependent, and was significant for all measured values when compared with the blank control group (*p* < 0.01). These results indicate that SNH can inhibit the synthesis of total protease, LasA staphylococcal protease, LasB elastase, LecA lectin, and pyocyanin, and inhibit and swimming ability in *P. aeruginosa*. Taken together, these results suggest that SNH may inhibit the activity of *P. aeruginosa* by inhibiting the expression of *P. aeruginosa* virulence factors.

### Effect of SNH on *P. aeruginosa* Biofilm Formation

**Figure 6:**
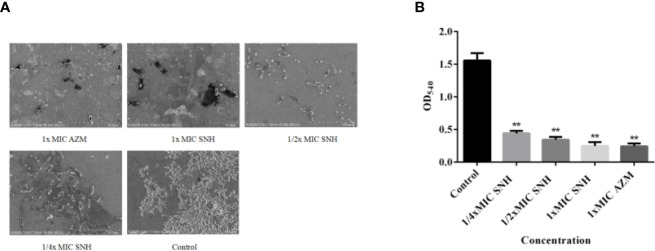
Sodium new houttuyfonate (SNH) effect on *P. aeruginosa* biofilm. **(A)** Morphology of ATCC 27853 biofilm under SEM (×5,000). **(B)** Effect of SNH on ATCC 27853 biofilm production. The drug concentration of treatments was as follows: Control, 512 μg/ml (1/4×MIC) SNH, 1,024 μg/ml (1/2×MIC) SNH, 2,048 μg/ml (1×MIC) SNH, and 64 μg/ml (1×MIC) azithromycin (AZM). Data represent the means ± SD (n=3, **p < 0.01 vs. control group).

*P. aeruginosa* causes chronic infection because it can form a biofilm. Therefore, inhibiting *P. aeruginosa* biofilm formation may be an effective way to inhibit chronic infection of *P. aeruginosa*. To explain the effect of SNH on *P. aeruginosa* biofilm, we used an inverted SEM and crystal violet staining. At 24 h, *P. aeruginosa* had begun the initial adhesion state. Control group bacteria gathered together in large numbers and had a mucus layer that surrounded a large number of bacteria. The biofilm treated by SNH gradually dispersed with increasing SNH concentration and the number of bacteria decreased (**Figure 6A**). The amount of biofilm formed in the control group was the largest. The amount of biofilm formed gradually decreased as the SNH concentration increased, showing a dose-dependent characteristic, and a significant difference was observed when compared with the blank control group (*p* < 0.01) (**Figure 6B**). These results confirm that SNH can inhibit the formation of *P. aeruginosa* biofilm.

### Effect of SNH on *P. aeruginosa* Gene Expression

**Figure 7:**
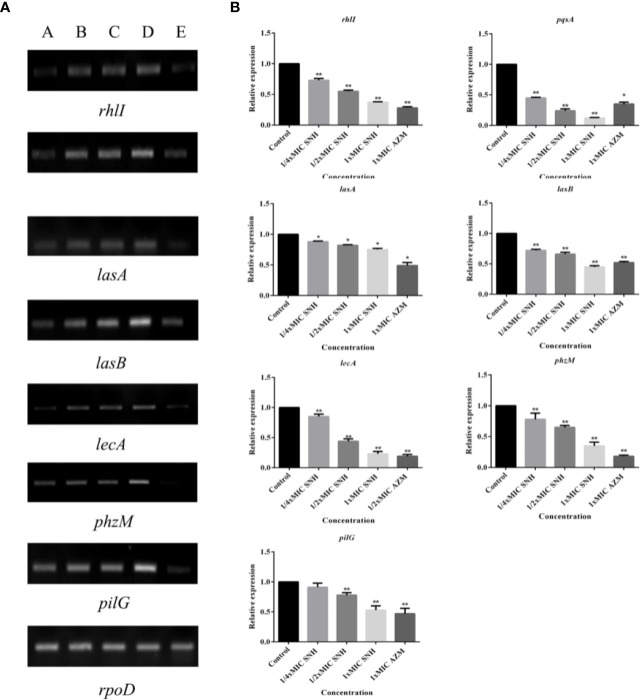
**(A)** RT-PCR results of sodium new houttuyfonate (SNH) inhibiting biofilm and related genes and virulence factors. The lanes from left to right were as follows: A: 2,048 μg/ml (1×MIC) SNH, B: 1,024 μg/ml (1/2×MIC) SNH, C: 512 μg/ml (1/4×MIC) SNH, D: Control, E: 1xMIC AZM. **(B)** qRT-PCR results of SNH inhibiting biofilm and related genes and virulence factors. Expression of the house-keeping gene, *rpoD*, was used as the internal control for each sample. The drug concentration of treatments was as follows: Control, 512 μg/ml (1/4×MIC) SNH, 1024 μg/ml (1/2×MIC) SNH, 2,048μg/ml (1×MIC) SNH, and 64 μg/ml (1×MIC) azithromycin (AZM). Data represent the means ± SD (n=3, *p < 0.05, **p < 0.01 vs. control group).

RT-PCR and qRT-PCR analysis were performed to verify gene expression in bacteria. The virulence genes, biofilms, and related genes controlled by the QS system were selected as the RT-PCR and qRT-PCR analysis targets. RT-PCR results showed that *lasA, lasB, lecA, rhlI, phzM, pqsA*, and *pilG* gene expression was down-regulated to different degrees after SNH treatment, and that the down regulation was dose dependent (**Figure 7A**). qRT-PCR results showed that increasing SNH concentration caused dose-dependent down regulation of the *lasA, lasB, lecA, rhlI, phzM, pqsA*, and *pilG* genes (**Figure 7B**). Although there are some differences in the RT-PCR, qRT-PCR, and RNA-seq results, the expression trends are basically the same. These results indicate that SNH can inhibit the expression of some virulence genes, and biofilm related genes regulated by the QS system. Therefore, SNH may inhibit *P. aeruginosa* pathogenicity by inhibiting the expression of virulence genes, biofilm genes, and related genes regulated by the QS system.

### *In Vivo* Evaluation of Efficacy of SNH Against *P. aeruginosa*

**Figure 8:**
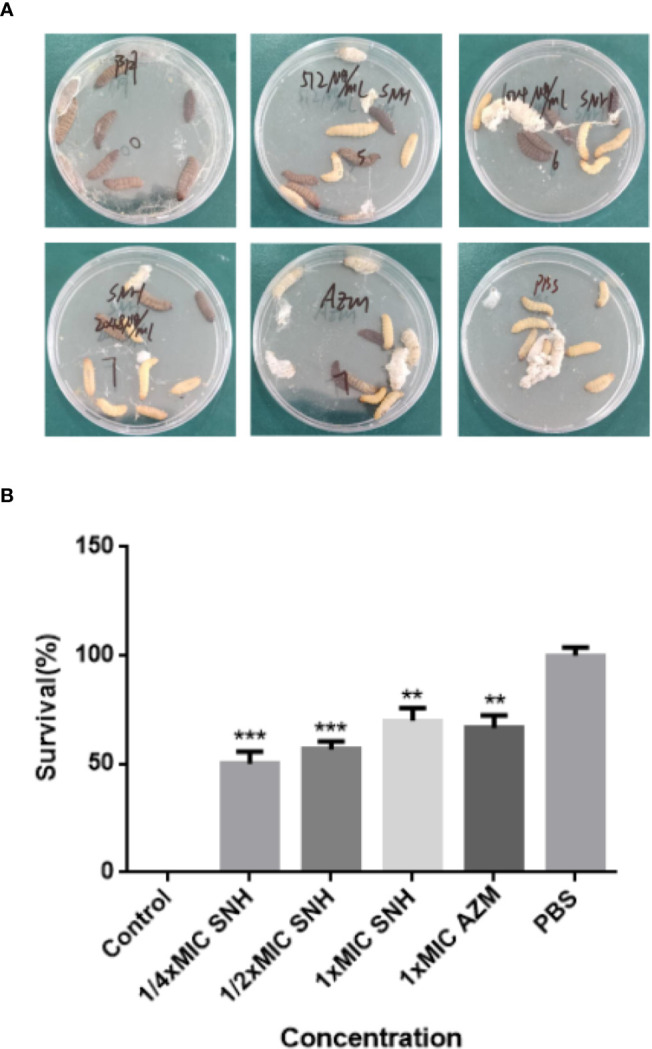
Survival rate of larvae infected by *P. aeruginosa*. **(A)** Survival status of larvae under different concentrations of SNH. **(B)** Survival rate of larvae at different concentrations of SNH. Data represent the means ± SD (n=3, **p < 0.01, ***p < 0.001 vs. control group).

After pre-experiment study, we determined 10^4 CFU per one larvae as the applied concentration LD_50_ of *G. mellonella* larvae. As shown in **Figure 8**, we investigated the effects of SNH treatment at 24 h post-infection of *P. aeruginosa* against *G. mellonella*. The results show that the survival rate of the infection + PBS group was 0%, and the infection + SNH 512 μg/mL group was 50%, and the infection + SNH 1024 μg/mL group was 60%, and the infection + SNH 2048 μg/mL group was 70%, and the infection + AZM 64 μg/mL group was 70%, and the PBS group was 100% at 24 h. These results also indicate that compared with untreated larvae, infected larvae showed significantly higher survival rate in drug treatment groups, and the difference was statistically significant (*p < 0.01*). Hence, these results suggest that SNH can improve the survival rate of larvae infected by *P. aeruginosa*, thereby effectively inhibit *P. aeruginosa* infection *in vivo*.

## Discussion

SH may play an antibacterial role by regulating the *P. aeruginosa* QS system to inhibit the synthesis of the virulence factors LasA protease and pyocyanin. SNH is a new generation product of Houttuynia cordata. Compared with SH, SNH has higher safety and effectiveness, is less toxic, has fewer side effects, and has better clinical promotion value (Yefei and Meixian, 2016). It has been recently reported that some fatty acids as antibiofilm and antivirulence agents (Kumar et al., 2020) as *Houttuyfonate* controlled QS-dependent phenotypes. The structures of *Houttuyfonate* and SNH are similar to long-chain fatty acids. Therefore, we speculate that SNH may also play an anti-*P. aeruginosa* effect based on QS-controlled signal transduction.

In order to determine the effect of SNH on the *P. aeruginosa* QS system and biofilm formation, we performed transcriptional analysis. Principle component analysis and correlation charts were used to evaluate the correlation between the blank control and SNH treatment groups (**Supplementary Material Figure 1**). Paired Pearson correlation coefficients for each group were higher than 0.7, proving that the samples were well correlated. Transcriptome analysis indicated that SNH may also play an antibacterial role by regulating the QS system of *P. aeruginosa*. Therefore, based on transcription analysis and experiments to examine the relationship between biofilm formation, the QS system, and pathogenicity, it may be possible to discover the mechanism of SNH action.

The virulence factors regulated by *P. aeruginosa* QS include protease, elastase, pyocyanin, lectin, rhamnolipid, toxin, and biofilm formation. These virulence factors can affect the formation and maintenance of biofilms and cluster movement. The mutual adjustment is complex, involving many internal and external environmental factors. Total protease can destroy the immunoglobulin that protects the mucous membrane, and destroy the tight junctions between host epithelial cells, which leads to invasion and injury of organism (Heras et al., 2014). Elastase is encoded by the lasB gene and plays an important role in *P. aeruginosa* infection, which can help bacteria damage host tissues and degrade immune proteins (Szamosvári et al., 2016). LecA is a galactose tetramer lectin produced by *P. aeruginosa*. It has affinity for extracellular polysaccharides, mediates the adhesion to host cells and infection and biofilm formation in *P. aeruginosa* (Huang et al., 2018). Pyocyanin is a blue-green redox secondary metabolite produced by *P. aeruginosa*, which can induce host neutrophils to accelerate apoptosis and reduce local inflammatory reaction, thus providing advantages for the survival of bacteria in the host. In the presence of biofilms, pyocyanin can induce the deposition of extracellular DNA (eDNA), which is the main component of biofilm extracellular polysaccharides and is essential for the formation and stability of biofilms. We further analyzed the effect of SNH on its virulence factors and biofilm formation. In *P. aeruginosa* biofilm, SNH affects the expression of a large number of genes. SNH reduced the synthesis of total protease, LasA staphylococcal protease, LasB elastase, LecA lectin, pyocyanin, and biofilm in *P. aeruginosa*. RT-PCR and qRT-PCR revealed that expression of QS regulated genes *rhlI, pqsA, lecA, lasA, lasB, phzM*, and *pilG* were down-regulated after SNH treatment.

In *P.aeruginosa*, SNH can inhibit the expression of some virulence factors and the formation of biofilms. Experimental results show that expression of these virulence genes, biofilms, and related genes is dose-dependent at concentrations of 1 x MIC SNH, ½ x MIC SNH and, ¼ x MIC SNH. Additionally, analysis of virulence factor, biofilm, and related gene expression was consistent with transcriptome sequencing and PCR analysis, further supporting that SNH has an inhibitory effect on *P. aeruginosa*.

Larval model is an important tool to study the pathogenesis of *P. aeruginosa* infection *in vivo*. We simulated the larval model, which proved that SNH significantly reduced the mortality of larvae. The difference of virulence between the drug groups and the blank control group proved by previous growth and virulence testing experiments can be confirmed in the larval model, which proves that the larval model is a suitable model for *P. aeruginosa* infection. The larval model can be used to study the pathogenic mechanism of *P. aeruginosa*. Once the genome sequence of larvae is available, the model of larvae will become more valuable in the future.

The MIC of SNH to *P. aeruginosa* was 2,048 μg/ml, which was four times higher than SH. This possible because our research group has been doing drug resistance tests on *P. aeruginosa* in recent years, which may gradually adapt to drugs and produce resistance. Previous studies found that SH could down-regulate *lasI* gene and up-regulate *rhlI* gene after 72 h of treatment by our research group. However, through transcriptome sequencing data, it was found that *lasI, rhlI*, and *pqsA* were down-regulated by 2.44, 1.5, and 3.78 times after SH treatment for 24 h in this study, while no change in *lasI* gene expression was detected after SNH treatment, and *rhlI* gene was down-regulated by 1.11 times and *pqsA* was down-regulated by 2.93 times, which was inconsistent with the previous research results, possibly due to the different action time selected in the experiment (72 h was selected in the previous experiment, while the action time selected in this experiment was 24 h). And it can be seen that when the action time is 24 h, the ways of SH and SNH on inhibiting *P. aeruginosa* are not completely the same. SH works together by regulating the Las/Rhl and PQS system, in which Las system plays a major role, while SNH plays a regulatory role by regulating Rhl and PQS system. However, due to the limitations of transcriptome sequencing, we also suspect that the relevant data may not be detected because of the small change multiple of *lasI*.

Importantly, we found that the expression of virulence genes at the transcription level is regulated in different ways among SNH and SH. Although *LasR* is considered as the main regulator in *P. aeruginosa*, the cross-regulation between the Las and Rhl system was observed in this experiment. For example, low but detectable C4-HSL is produced without LasR (Wang et al., 2018). The specific reason the expression of *lasR* is not detected in SNH is still unclear, which may be caused by other highly interactive QS regulatory systems, such as Vfr, VqsR, MvaT, GacA, RpoN, RpoS, and RsmA (Balasubramanian et al., 2013). The existence of Rhl signal without Las signal in clinical isolates suggests that las-independent QS activity may play a role in maintaining *P. aeruginosa* infection (Feltner et al., 2016). There are researchers reported that *P. aeruginosa* may enhance some virulence genes and reduce the expression of other genes as part of its survival strategy (Wang et al., 2018). We observed that the yield of total protease and lasB decreased in this experiment, which may be related to the high level of Psl. Therefore, exploring whether the presence of Psl levels in SNH is related to *P. aeruginosa* infection would be an interesting research direction in the future.

Our research shows that further study on QS system of *P. aeruginosa* may be helpful to understand its pathogenic mechanism. Nevertheless, there was no change of lasR gene in ATCC 27853 under the action of SNH, which indicated that Las system was not absolutely necessary for the establishment of *P. aeruginosa* infection. In addition, our study also questioned the previously recognized level understanding of the QS system, and the mechanism should be understood more comprehensively in many aspects and combination with specific situations i.e., different strains and hosts.

Combined with the above experiments, we infer that these virulence factors and biofilms are inhibited by SNH inhibiting the production of C4-HSL. The difference of action mechanism between SH and SNH on *P. aeruginosa* may lie in the number of C atoms in its structure, which is more than SH 2 C atoms in SNH, which may cause SNH not combining with 3-oxo-C12-HSL, combining with RhlR and C4-HSL complex to play an antibacterial role. SNH has replaced houttuynin, and to a large extent SH due to its improved chemical and pharmacological properties, which has been used as an effective therapeutic agent for respiratory infections and inflammatory diseases such as acute or chronic bronchitis and pneumonia (Lu et al., 2013). Through the experimental study, it was found that SNH had a certain inhibitory effect on *P. aeruginosa*, but its MIC was high, which provided a certain basis for its structural modification and transformation in the future.

## Conclusion

**Figure 9:**
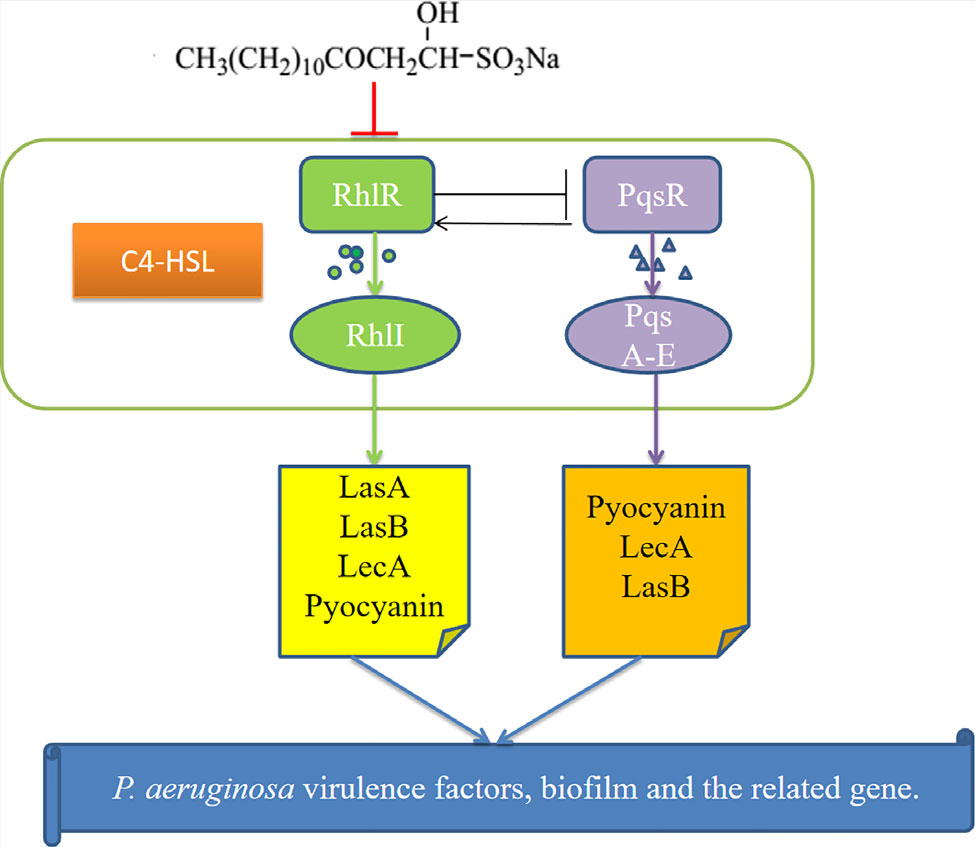
Possible mechanism for the inhibition of *P.aeruginosa* quorum sensing (QS) system by sodium new houttuyfonate (SNH). The figure shows the Rhl and PQS systems and the virulence genes regulated by them. Our results show that SNH inhibits the expression of *rhlI*, and *pqsA*. The scheme shows quorum sensing(QS)-controlled gene regulation, Rhl can produce virulence factors LasA, elastase B, lectin LecA, pyocyanin, etc. Alone or in cooperation with PQS. These results confirm that SNH interferes with the production of QS and QS-related virulence factors of *P.aeruginosa*. Therefore, the pathogenicity of *P.aeruginosa* may be weakened by inhibiting the formation of virulence factors and biofilm.

These results show that SNH may inhibit the *P. aeruginosa* QS system, reduce the synthesis of virulence factors and biofilms, and down-regulate the expression of key genes (**Figure 9**) to inhibit *P. aeruginosa* infection. Additionally, this study can be used as a basis for designing new QS-targeted drug candidates using SNH, while minimizing the chance of generating resistance (Rodolfo et al., 2013). These results are currently being expanded upon through research on animal infection models *in vivo* in our research group.

## Data Availability Statement

The original contributions presented in the study are included in the article/**Supplementary Material**; further inquiries can be directed to the corresponding author.

## Author Contributions

DW, GY, and CW conceived and designed the study. YZ and DW wrote the manuscript. JS and TW critically reviewed the manuscript and provided general advice. YZ, LM, YS, and JW performed the experiments and analyzed data. All authors contributed to the article and approved the submitted version.

## Funding

This work was supported by the project funded by the China Postdoctoral Science Foundation under Grant No. 2019M662185, the key discipline of Anhui University of Chinese Medicine (DC18100042), the Outstanding Talent Support Program in University (Key project) of Anhui Province under Grant No. gxyqZD2020024, and the Natural Science Foundation (Key project) of Anhui University of Chinese Medicine under Grant No.2020zrzd07. The Funding is used to cover laboratory expenses, sample preparation and sequencing. These funding agencies have no role in research design, data collection, analysis of results, or manuscript writing.

## Conflict of Interest

The authors declare that the research was conducted in the absence of any commercial or financial relationships that could be construed as a potential conflict of interest.

## Supplementary Material

Supplementary Figure 1 (A) PCA and (B) Correlation coefficient diagram between SNH treated group and control group.

Supplementary Figure 2 The bacterial secretion system Type I, Type II, Type III, and Type VI was changed under SNH treatment.
