# Supplementary figures and images for: Sodium New Houttuyfonate Affects Transcriptome and Virulence Factors of Pseudomonas aeruginosa Controlled by Quorum Sensing
Source: Front Pharmacol. 2020 Oct 2;11:572375. doi: 10.3389/fphar.2020.572375 (PMC7566558; doi:10.3389/fphar.2020.572375)

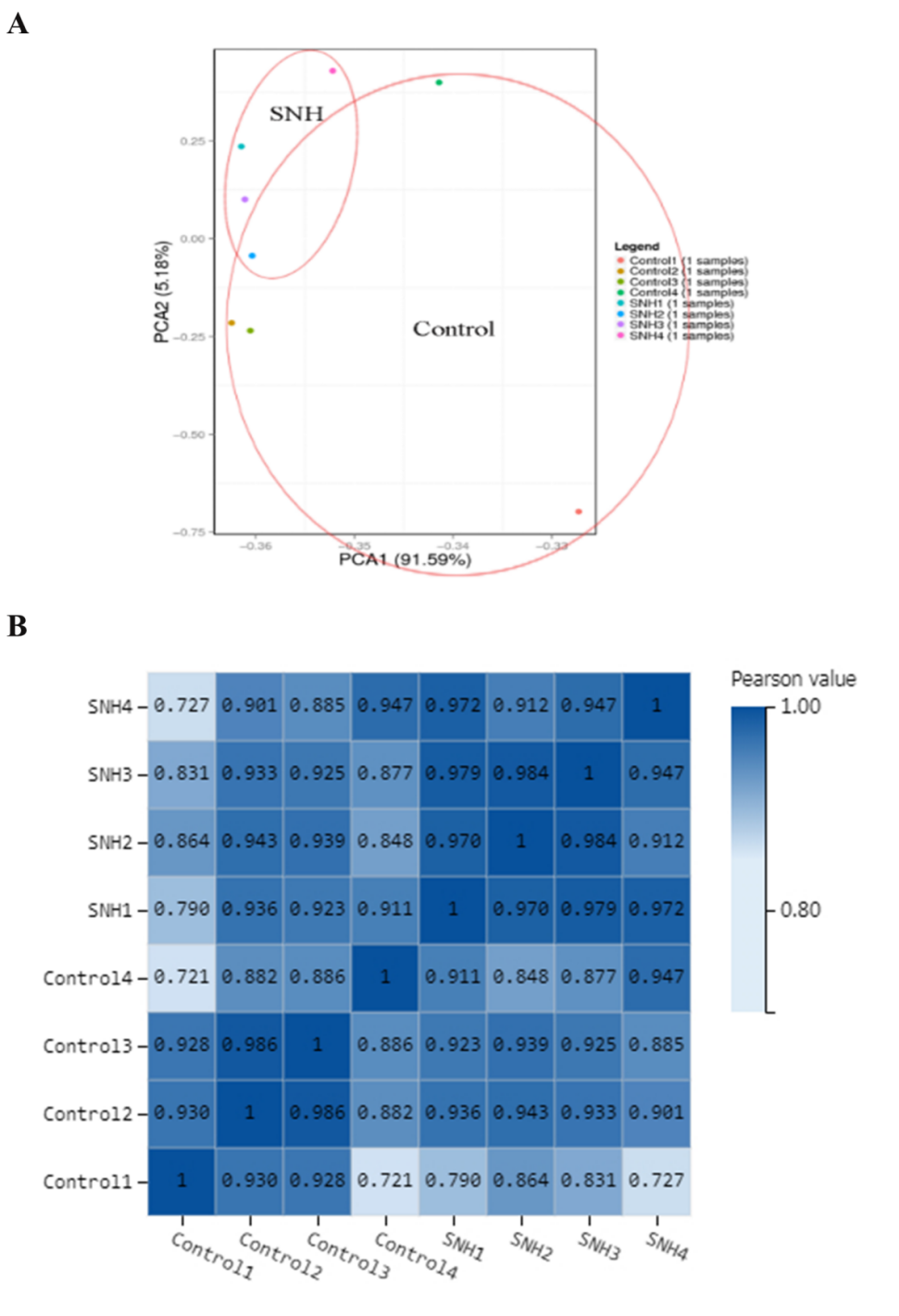

Supplement: Supplementary Figure 1 — (A) PCA and (B) Correlation coefficient diagram between SNH treated group and control group. [file Image_1.tif]

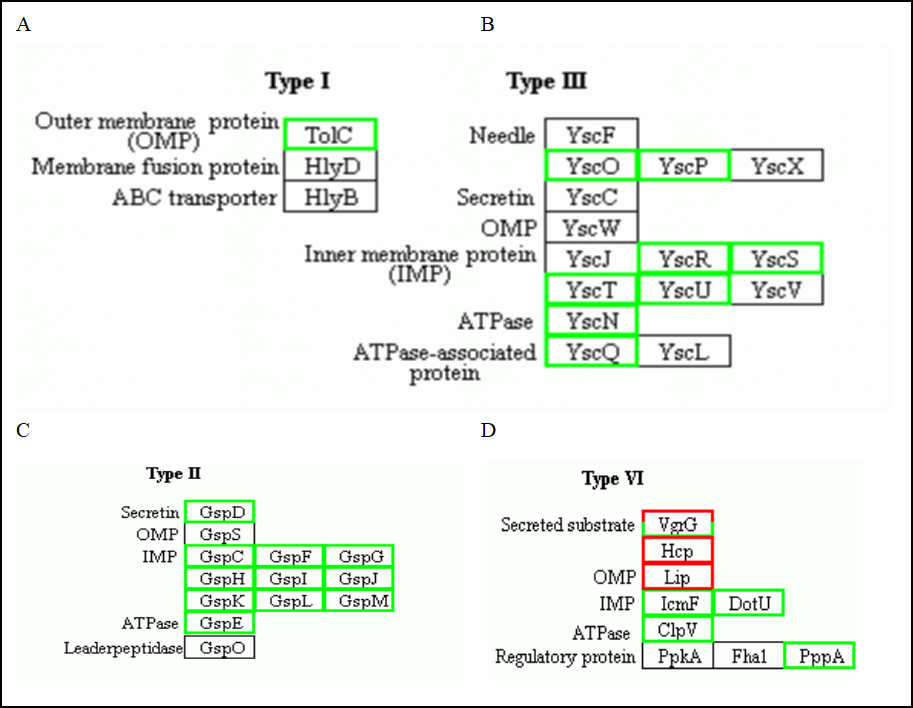

Supplement: Supplementary Figure 2 — The bacterial secretion system Type I, Type II, Type III, and Type VI was changed under SNH treatment. [file Image_2.tif]
